# Supplementary material for: Wearable devices for anxiety assessment: a systematic review
Source: Commun Med (Lond). 2026 Jan 9;6:20. doi: 10.1038/s43856-025-01234-6 (PMC12789550; doi:10.1038/s43856-025-01234-6)
Supplement: Supplementary file 2 — Description of Additional Supplementary Files [file 43856_2025_1234_MOESM2_ESM.docx]

**Description of Additional Supplementary Files**

File name: Supplementary Data 1

Description: Comparison of single-modality studies

File name: Supplementary Data 2

Description: Comparison of multi-modality studies
