# Supplementary material for: Wearable devices for anxiety assessment: a systematic review
Source: Commun Med (Lond). 2026 Jan 9;6:20. doi: 10.1038/s43856-025-01234-6 (PMC12789550; doi:10.1038/s43856-025-01234-6)
Supplement: Supplementary file 3 — Supplementary Data 1 [file 43856_2025_1234_MOESM3_ESM.pdf]

Supplementary Data 1 Comparison of single-modality studies

Signal[a]	Study	Anxiety induction[b]	Anxiety measurement[c]	Anxiety labeling approach	WD (body part)[d]	Sample size	Age [years][e]	Features[f]	Method[g]	Validation[h]	Result[i]
ECG	Jain & Kumar, 2024[1]	TSST	6-STAI	3-class (low/moderate/high) based on 6-STAI scores	RespiBAN (chest)	15 (M: 12, F: 3)	27.5 ± 2.4 (25–29)	Min ECG, Max ECG, Mean ECG, Var ECG, SD ECG, Mean HR, Mean RR, RMSSD, SDNN, TINN	GBT	LOSO CV	ACC = 89.80%
ECG	Baygin et al., 2024[2]	Videos	HAM	4-class (normal/mild/moderate/severe) based on HAM scores	SS2LB ECG module (both wrists, right leg)	19 (M: 14, F: 5)	26.2 ± 8.7 (18–56)	PBP-based features (spatial + frequency), reduced to 256 features via NCA and Chi2.	SVM + CMV	10-fold CV	ACC = 99.95%, F1 = 99.94%
ECG	Sinche et al., 2024[3]	Pre- and post-test evaluation	Cognitive Test Anxiety Scale	Binary (anxious vs. non-anxious) based on pre-test (anxious) vs. post-test (non-anxious) phases	AD8232 sensor (3 body electrodes)	20 (M: 18, F: 2)	19–27	Mean HR, SDNN, RMSSD, pNN50, SDSD	SVM	10-fold CV	ACC = 84.51%
ECG	Tripathy et al., 2023[4]	Videos	HAM	4-class (normal/mild/moderate/severe) based on HAM scores	SS2LB ECG module (both wrists, right leg)	19 (M: 14, F: 5)	26.2 ± 8.7 (18–56)	Increment entropy and energy of 100 FBDAWT modes, forming a 200-D feature vector	XGB	Hold-out (N/R)	ACC = 92.27%, SE = 92.54%, PPV = 92.02%, F1 = 92.13%
ECG	Padmaja et al., 2023[5]	N/R	N/R	Binary (anxious vs. non-anxious), binarization method not specified	Biopac MP-45 system (N/R)	500 (N/R)	20–30	N/R	N/R	N/R	ACC = 96.00%
ECG	Gazi et al., 2021[6]	VRET spider exposure	Self-reported anxiety level	Binary (anxious vs. rest) based on exposure to spider clips vs. rest condition	BITalino (both wrists, chest)	55 (N/R)	18–40	Mean HR, SD HR, NFD HR, Mean RR, SDNN, RMSSD, pNN50, LF/HF, SD1/SD2, RSA	RF	LOSO CV	ACC = 60.00%, F1 = 59.00%
ECG	Vulpe-Grigorasi et al., 2021[7]	VRET spider exposure	Self-reported anxiety level	Binary (anxious vs. rest) based on exposure to spider clips vs. rest condition	BITalino (both wrists)	57 (N/R)	18–40	RMSSD (10 – 60 s intervals)	1D CNN Model	Hold-out (train 80%, validation 10%, test 10%)	ACC = 77.14%, SE = 82.00%, PPV = 85.00%
ECG	Tang et al., 2021[8]	N/R	N/R	Binary (anxious vs. normal), binarization method not specified	N/R	6 (N/R)	N/R	VLF Power, LF Power, HF Power	DBSCAN-enhanced algorithm	N/R	ACC = 72.65%
ECG	Wen et al., 2020[9]	TSST	Self-reported anxiety level and audience scores	Binary (anxious vs. rest) based on TSST (anxious) vs. baseline resting state	MP150 recorder (both wrists, right ankle)	42 (N/R)	17–20	SDNN, LF power, HF power, pNN50, SD FDRR, SD aFDRR, Mean FDRR, Mean aFDRR, Mean aFDnRR, Mean aFSnRR, Mean RR, Hurst Exp Range	SVM	5-fold CV	ACC = 91.60%, SE = 84.88%, SP = 97.29%, PPV = 96.37%, F1 = 90.09%
ECG	Ismail et al., 2020[10]	Experimental SAD Task	DSM-5, LSAS	N/R	N/R	34 (M: 13, F: 21)	20–27	Mean HR	DT	N/R	ACC = 88.90%
ECG	Tiwari et al., 2019[11]	Hospital work	Daily self-reported 5-point scale	Binary (high vs. low anxiety) based on 5-point daily self-ratings, binarization method not specified	OMsignal smartshirt (N/R)	196 (M: 66, F: 130)	38.6 ± 9.8	Mean RR, SDNN, RMSSD, pNN50, SD aFDRR, Mean FDRR, Mean aFDnRR, Total power, LF Power, HF Power, VLF Power, LF/HF, HF Norm, LF Norm	SVM	5-fold CV	ACC = 57.65%, SE = 51.95%, SE = 63.35%, F1 = 49.06%
ECG	Tiwari et al., 2019[12]	Hospital work	Daily self-reported 5-point scale	Binary (high vs. low anxiety) based on 5-point daily self-ratings, binarization method not specified	OMsignal smartshirt (N/R)	200 (M: 66, F: 134)	38.6 ± 9.8	Mean RR, SDNN, RMSSD, pNN50, SD aFDRR, Mean FDRR, Mean aFDnRR, LF Power, HF Power, VLF Power, HF/LF, HF Norm, LF Norm, MSPE, MSmPE, dRR_MSPE, dRR_MSmPE, Pedw, Total Asymmetry Index, dRR_MotifDiff_s1, dRR_MotifDiff_s2, dRR_MotifDiffSum_s1, dRR_MotifDiffSum_s2	SVM	5-fold CV	ACC = 62.77%, SE = 61.09%, SP = 64.45%, F1 = 55.78%

(Table continued)

Signal[a]	Study	Anxiety induction[b]	Anxiety measurement[c]	Anxiety labeling approach	WD (body part)[d]	Sample size	Age [years][e]	Features[f]	Method[g]	Validation[h]	Result[i]
EDA	Jain & Kumar, 2024[1]	TSST	6-STAI	3-class (low/moderate/high) based on 6-STAI scores	RespiBAN (chest)	15 (M: 12, F: 3)	27.5 ± 2.4 (25–29)	Min EDA, Max EDA, Mean EDA, SD EDA, Mean Peak Duration SCR, Mean Peak Amp SCR	GBT	LOSO CV	ACC = 85.90%
EDA	Nath and Thapliyal, 2021[13]	TSST	STAI	Binary (anxious vs. non-anxious) based on STAI scores above/below population mean	Wearable wristband (N/R)	41 (M: 15, F: 26)	73.4 ± 5.3 (60–80)	Mean Peak Amp SCR, Median Peak Amp SCR, SD Peak Amp SCR, RMS Peak Amp SCR, Max Peak AMP SCR, Min Peak Amp SCR, Mean Peak Width SCR, Median Peak Width SCR, SD Peak Width SCR, RMS Peak Width SCR, Max Peak Width SCR, Min Peak Width SCR, Mean Peak Prom SCR, Median Peak Prom SCR, SD Peak Prom SCR, RMS Peak Prom SCR, Max Peak Prom SCR, Min Peak Prom SCR	RF	Hold-out (train 50%, validation 10%, test 40%)	ACC = 89.00%, F1 = 86.00%
EDA	Lee et al., 2020[14]	Videos	Self-reported anxiety onsets	Binary (anxious vs. non-anxious) based on immediate self-report after each video	Empatica E4 (wrist)	23 (N/R)	23.3 ± 1.9	Mean EDA, SD EDA, Max EDA, Min EDA, Amp EDA	LR	LOTO CV	ACC = 42.53%
EDA	Gazi et al., 2021[6]	VRET spider exposure	Self-reported anxiety level	Binary (anxious vs. rest) based on exposure to spider clips vs. rest condition	BITalino (both wrists, chest)	55 (N/R)	18–40	Mean SCL, NFD SCL, Nr of Peaks SCR, Mean Peak Amp SCR	RF	LOSO CV	ACC = 87.00%, F1 = 87.00%
EDA	Shaukat-Jali et al., 2021[15]	Impromptu speech task	LSAS, SPSQ	Binary (anxious vs. baseline) based on experimental stage timestamps	Empatica E4 (wrist)	12 (M: 5, F: 7)	19 ± 1.8	N/R	KNN	10-fold CV	ACC = 80.46%
EDA	Šalkevičius et al., 2019[16]	VRET public speaking	SUDS (0-100 scale)	4-class (low/mild/moderate/high) based on SUDS score	Empatica E4 (wrist)	30 (M: 17, F: 13)	27.5 ± 4.2 (21–34)	Min EDA, Max EDA, Mean EDA, Var EDA, SD EDA, Median EDA, Kurt EDA, Skew EDA, MAD EDA, 6th Mom EDA, 5th Mom EDA, 4th Mom EDA, 3rd Mom EDA, RMS EDA, 1st Dif EDA, 1st Dif/SD EDA, 2nd Dif EDA , 2nd Dif /SD EDA, MA Raw EDA, SD Raw EDA, MA 1st Dif Raw EDA, MA 1st Dif norm EDA, MA 2nd Dif EDA, MA 2nd Dif norm EDA, Nr of Peaks SCR, Mean Peak Amp SCR, Max Peak Amp SCR	SVM	10-fold CV	ACC = 76.60%
PPG	Bao et al., 2024[17]	Rest and relaxation with biofeedback	STAI	3-class (relaxed/mild/severe) based on STAI score	Empatica E4 (wrist)	20 (M: 0, F: 20)	29.1 ± 3.6	Mean HR, Mean PPG, SD PPG, MSE PPG, LF Power PPG, MF Power PPG, HF Power PPG, Spectral Power Ratio PPG, Tachogram Spectral Values, Tachogram Spectral Ratio, SDNN, LF/HF, RMSSD, PNSi, SNSi	SVM	LOSO CV	F1 = 64.20%
PPG	Nath and Thapliyal 2021[13]	TSST	STAI	Binary (anxious vs. non-anxious) based on STAI scores above/below population mean	Wearable wristband (N/R)	41 (M: 15, F: 26)	73.4 ± 5.3 (60–80)	Mean HR, Mean Peak Width PPG, Median Peak Width PPG, SD Peak Width PPG, RMS Peak Width PPG, Max Peak Width PPG, Min Peak Width PPG, Mean Peak Prom PPG, Median Peak Prom PPG, SD Peak Prom PPG, RMS Peak Prom PPG, Max Peak Prom PPG, Min Peak Prom PPG, Mean Peak Amp PPG, SD Peak Amp PPG, RMS Peak Amp PPG, Range Peak Amp PPG	RF	Hold-out (train 50%, validation 10%, test 40%)	ACC = 78.00%, F1 = 71.00%

(Table continued)

Signal[a]	Study	Anxiety induction[b]	Anxiety measurement[c]	Anxiety labeling approach	WD (body part)[d]	Sample size	Age [years][e]	Features[f]	Method[g]	Validation[h]	Result[i]
PPG	Lee et al., 2020[14]	Videos	Self-reported anxiety onsets	Binary (anxious vs. non-anxious) based on immediate self-report after each video	Empatica E4 (wrist)	23 (N/R)	23.3 ± 1.9	Mean Amp PPG, SD Amp PPG, Max Amp PPG, Min Amp PPG, Mean Dif PPI, SD Dif PPI, Length Dif PPI, Irregularity Dif PPI, Dif nPPI, Post Count Dif Fast PPI, LF/HF, CoV PPI	LR	LOTO CV	ACC = 49.75%
PPG	Sinche et al., 2024[3]	Pre- and post-test evaluation	Cognitive Test Anxiety Scale	Binary (anxious vs. non-anxious) based on pre-test (anxious) vs. post-test (non-anxious) phases	MAX30100 (finger)	20 (M: 18, F: 2)	19–27	SDNN, RMSSD, pNN50, Mean HR, Mean PPI	SVM	10-fold CV	ACC = 64.14%
PPG	Shaukat-Jali et al., 2021[15]	Impromptu speech task	LSAS, SPSQ	Binary (anxious vs. baseline) based on experimental stage timestamps	Empatica E4 (wrist)	12 (M: 5, F: 7)	19 ± 1.8	N/R	KNN	10-fold CV	ACC = 68.18%
PPG	Šalkevičius et al., 2019[16]	VRET public speaking	SUDS (0-100 scale)	4-class (low/mild/moderate/high) based on SUDS score.	Empatica E4 (wrist)	30 (M: 17, F: 13)	27.5 ± 4.2 (21–34)	Min PPG, Max PPG, Mean PPG, Var PPG, SD PPG, Median PPG, Kurt PPG, Skew PPG, MAD PPG, 6th Mom PPG, 5th Mom PPG, 4th Mom PPG, 3rd Mom PPG, RMS PPG, 1st Dif PPG, 1st Dif/SD PPG, 2nd Dif PPG , 2nd Dif /SD PPG, MA Raw PPG, SD Raw PPG, MA 1st Dif Raw PPG, MA 1st Dif norm PPG, MA 2nd Dif PPG, MA 2nd Dif norm PPG, Mean HR, MAD HR, RMSSD, SDNN, Mean PPI, SD PPI	SVM	10-fold CV	ACC = 74.10%
RSP	Gazi et al., 2021[6]	VRET spider exposure	Self-reported anxiety level	Binary (anxious vs. rest) based on exposure to spider clips vs. rest condition	BITalino (both wrists, chest)	55 (N/R)	18–40	Mean ReR, SD ReR, Mean IBI, SD IBI, RMSSD ReR, CoV ReR, ACF1 of ReR, CoV Ti, ACF1 Ti, RMSSD Ti, CoV Te, ACF1 Te, RMSSD Te, Mean Ti, Mean Te, Mean Ti/Te	RF	LOSO CV	ACC = 53.00%, SE = 67.00%
RSP	Tiwari et al., 2019[18]	Hospital work	Daily self-reported 5-point scale	Binary (high vs. low anxiety) from 5-point daily self-ratings (binarization not specified)	OMsignal smartshirt (N/R)	200 (M: 66, F: 134)	38.6 ± 9.8	Mean ReR, SD ReR, Mean BD, SD BD, Mean Ti/Te, SD Ti/Te, CoV Ti/Te, PE Ti/Te, SampEn Ti/Te, dCor Ti/Te, Mean Modulation (Ti/Te)/RR, SD Modulation (Ti/TE)/RR, Mean IBI, Mean IBI, CoV IBI, PE IBI, SampEn IBI, dCor IBI, Mean IBI(d), CoV IBI(d), PE IBI(d), SampEn IBI(d), dCor IBI(d), Mean Ai, SD Ai, CoV Ai, Mean Ae, SD Ae, CoV Ae	SVM	5-fold CV	ACC = 62.39%, SE = 54.97%, SP = 61.20%, F1 = 63.59%
RSP	Banerjee et al., 2019[19]	Playing affective Pacman game	SAM	Binary (anxious vs. normal) based on trial condition (standard vs affective Pacman), and supported by SAM rating	Respiration belt (abdomen)	10 (N/R)	N/R	Mean Ti, Mean Te, BPM, Tv, Pmax, Pmin, Ri, Re, Mean Ti/Te, Vi, Ve, Vm, Stretch	MLP	10-fold CV	ACC = 97.90%, SE = 92.60%, SP = 99.30%
RSP	Haritha et al., 2017[20]	No task	Clinical psychiatric diagnosis	Binary (anxious vs. normal) based on clinical diagnosis	Embletta (N/R)	41 (M: 25, F: 16)	N/R	Mean IBI, Median IBI, SDNN IBI, SDANN IBI, NNx, pNNx, RMSSD IBI, SDNNi IBI, Mean ReR, SD ReR, HRVTi, TINN, VLF Power, LF Power, HF Power	SVM	N/R	ACC = 92.30%

Notes:

[a] ECG = electrocardiogram, EDA = electrodermal activity, PPG = photoplethysmogram, RSP = respiratory signal.

[b] TSST = Trier Social Stress Test, SAD = Social Anxiety Disorder, VRET = Virtual Reality Exposure Therapy, N/R = not reported.

[c] 6-STAI = Six-Item State-Trait Anxiety Inventory, HAM = Hamilton Anxiety Rating Scale, DSM-5 = Diagnostic and Statistical Manual of Mental Disorders, 5th edition, LSAS = Liebowitz Social Anxiety Scale, SAS-SR = Social Avoidance and Distress Scale - self report, SPSQ = Social Phobia Screening Questionnaire, SUDS = Subjective Units of Distress Scale, SAM = Self-Assessment Manikin.

[d] WD = wearable device

[e] Min = minimum, Max = maximum, Var = variance, SD = standard deviation, HR = heart rate, RR = R-R interval (time between successive R waves in an ECG signal), RMSSD = root mean square of successive differences of R-R intervals (or pulse peak intervals (PPI) in PPG), SDNN = standard deviation of normal-to-normal R-R intervals (or PPI in PPG), TINN = triangular interpolation of NN interval histogram, PBP = probabilistic binary pattern, NCA = neighborhood component analysis, Chi2 = Chi-square, pNN50 = percentage of successive R-R intervals differing by more than 50 ms, SDSD = standard deviation of successive differences of R-R intervals, FBDAWT = fixed-band discrete approximation wavelet transform, NFD = normalized first difference, LF = low frequency, HF = high frequency, SD1/SD2 = Poincaré plot features: short-term (SD1) and long-term (SD2) variability, RSA = respiratory sinus arrhythmia, VLF = very low frequency (0.003–0.04 Hz), FDRR = first differences of R-R intervals, aFDRR = absolute first differences of R-R intervals, aFDnRR = absolute first differences of normalized R-R intervals, aFSnRR = absolute second differences of normalized R-R intervals, Exp = exponent, Norm = normalized, MSPE = multi-scale permutation entropy, MSmPE = multi-scale modified permutation entropy, dRR = differential R-R interval series, Pedw = peak detection windowing, MotifDiff (s1, s2) = differences in motif distributions between scale 1 and scale 2, MotifDiffSum = summed motif distribution differences, SCR = skin conductance response (phasic component of EDA), Amp = amplitude, Prom = prominence (of signal peaks), SCL = skin conductance level (tonic component of EDA), Nr = number, N/R = not reported, Kurt = kurtosis, Skew = skewness, MAD = mean absolute deviation, Mom = moment (3rd, 4th, 5th, or 6th moment of the signal distribution), Dif = difference, MA = mean absolute value, MSE = multi-scale entropy, PNSi = parasympathetic nervous system index, SNSi = sympathetic nervous system index, CoV = coefficient of variation, PPI = pulse peak interval, ReR = respiratory rate, IBI = inter-breath interval, ACF1 = first-lag autocorrelation, Ti = inspiratory time, Te = expiratory time, TBD = tidal breathing depth, PE = permutation entropy, SampEn = sample entropy, dCor = correlation dimension, IBI(d) = inter-breath interval difference, Ai = inspiratory amplitude, Ae = expiratory amplitude, Tv = tidal volume, Pmax = maximum inspiratory flow, Pmin = minimum expiratory flow, Ri = inspiratory ratio, Re = expiratory ratio, Vi = inspiratory volume, Ve = expiratory volume, Vm = minute ventilation volume, SDANN = standard deviation of averages of NN intervals over short periods, NNx = number of successive differences greater than x ms, pNNx = percentage of successive differences greater than x ms, SDNNi = standard deviation of NN intervals for each short interval, HRVTi = heart rate variability for time intervals.

[f] GBT = gradient boosted trees, SVM = support vector machine, CMV = combinational majority voting, XGB = extreme gradient boosting, RF = random forest, CNN = convolutional neural network, DBSCAN = density-based spatial clustering of applications with noise, DT = decision trees, LR = logistic regression, KNN = k-nearest neighbor, MLP = multi-layer perception.

[g] LOSO = leave-one-subject-out, CV = cross-validation, LOTO = leave-one-trial-out.

[h] ACC = accuracy, F1 = F1 score, PPV = precision, SE = sensitivity, SP = specificity, ACC = estimated accuracy, F1 = estimated F1 score, PPV = estimated precision, SE = estimated sensitivity, SP = estimated specificity

References

1. Jain, A. & Kumar, R. Machine learning based anxiety detection using physiological signals and context features. In 2024 2nd International Conference on Advancement in Computation & Computer Technologies (InCACCT), 116–121, DOI: 10.1109/InCACCT61598.2024.10551003 (2024).
2. Baygin, M. et al. Automated anxiety detection using probabilistic binary pattern with ECG signals. Comput. Methods Programs Biomed. 247, 108076, DOI: 10.1016/j.cmpb.2024.108076 (2024).
3. Sinche, S., Acán, J. & Hidalgo, P. Anxiety detection using consumer heart rate sensors. Eng. Proc. 77, DOI: 10.3390/engproc2024077010 (2024).
4. Tripathy, R. K., Dash, D. K., Ghosh, S. K. & Pachori, R. B. Detection of different stages of anxiety from single-channel wearable ECG sensor signal using Fourier–Bessel domain adaptive wavelet transform. IEEE Sensors Lett. 7, 1–4, DOI: 10.1109/LSENS.2023.3274668 (2023).
5. Padmaja, K. V. et al. Anxiogram: Unmasking anxiety with IoT-enhanced ECG. In 2023 7th International Conference on Computation System and Information Technology for Sustainable Solutions (CSITSS), 1–4, DOI: 10.1109/CSITSS60515.2023.10334100 (2023).
6. Gazi, A. H. et al. Respiratory markers significantly enhance anxiety detection using multimodal physiological sensing. In 2021 IEEE EMBS International Conference on Biomedical and Health Informatics (BHI), 1–4, DOI: 10.1109/BHI50953.2021.9508589 (Athens, Greece, 2021).
7. Vulpe-Grigoraşi, A. & Grigore, O. A neural network approach for anxiety detection based on ecg. In 2021 International Conference on e-Health and Bioengineering (EHB), 1–4, DOI: 10.1109/EHB52898.2021.9657544 (Iasi, Romania, 2021).
8. Tang, Y., Zhou, N. & Guan, A. Anxiety detection algorithm for wearable devices based on DBSCAN. In 2021 6th International Conference on Intelligent Computing and Signal Processing (ICSP), 56–60, DOI: 10.1109/ICSP51882.2021.9408868 (2021).
9. Wen, W. et al. Toward constructing a real-time social anxiety evaluation system: Exploring effective heart rate features. IEEE Transactions on Affect. Comput. 11, 100–110, DOI: 10.1109/TAFFC.2018.2792000 (2020).
10. Ismail, N. M., Airij, A. G., Sudirman, R. & Omar, C. Early detection of social anxiety disorder by using screening tools and wearable sensors. In 2020 6th International Conference on Computing Engineering and Design (ICCED), 1–6, DOI: 10.1109/ICCED51276.2020.9415828 (2020).
11. Tiwari, A., Cassani, R., Narayanan, S. & Falk, T. H. A comparative study of stress and anxiety estimation in ecological settings using a smart-shirt and a smart-bracelet. In 2019 41st Annual International Conference of the IEEE Engineering in Medicine and Biology Society (EMBC), 2213–2216, DOI: 10.1109/EMBC.2019.8857890 (2019).
12. Tiwari, A., Narayanan, S. & Falk, T. H. Stress and anxiety measurement "in-the-wild" using quality-aware multi-scale HRV features. In 2019 41st Annual International Conference of the IEEE Engineering in Medicine and Biology Society (EMBC), 7056–7059, DOI: 10.1109/EMBC.2019.8857616 (Berlin, Germany, 2019).
13. Nath, R. & Thapliyal, H. Machine learning-based anxiety detection in older adults using wristband sensors and context feature. SN Comput. Sci. 2, 359, DOI: 10.1007/s42979-021-00744-z (2021).
14. Lee, S., Lee, T., Yang, T., Yoon, C. & Kim, S.-P. Detection of drivers' anxiety invoked by driving situations using multimodal biosignals. Processes 8, DOI: 10.3390/pr8020155 (2020).
15. Shaukat-Jali, R., van Zalk, N. & Boyle, D. Detecting subclinical social anxiety using physiological data from a wrist-worn wearable: Small-scale feasibility study. JMIR Form. Res. 5, e32656, DOI: 10.2196/32656 (2021).
16. Šalkevicius, J., Damaševičius, R., Maskeliunas, R. & Laukienė, I. Anxiety level recognition for virtual reality therapy system using physiological signals. Electronics 8, DOI: 10.3390/electronics8091039 (2019).
17. Bao, Y., Xue, M., Gohumpu, J. et al. Prenatal anxiety recognition model integrating multimodal physiological signal. Sci. Reports 14, 21767, DOI: 10.1038/s41598-024-72507-8 (2024).
18. Tiwari, A., Narayanan, S. & Falk, T. H. Breathing rate complexity features for "in-the-wild" stress and anxiety measurement. In 2019 27th European Signal Processing Conference (EUSIPCO), 1–5, DOI: 10.23919/EUSIPCO.2019.8902700 (2019).
19. Banerjee, T., Khasnobish, A., Chowdhury, A. & Chatterjee, D. Reckoning respiratory signals to affectively decipher mental state. In 2019 41st Annual International Conference of the IEEE Engineering in Medicine and Biology Society (EMBC), 4654–4659, DOI: 10.1109/EMBC.2019.8857498 (Berlin, Germany, 2019).
20. Haritha, H., Negi, S., Menon, R. S., Kumar, A. A. & Kumar, C. S. Automating anxiety detection using respiratory signal analysis. In 2017 IEEE Region 10 Symposium (TENSYMP), 1–5, DOI: 10.1109/TENCONSpring.2017.8069995 (2017).
