# Supplementary material for: Wearable devices for anxiety assessment: a systematic review
Source: Commun Med (Lond). 2026 Jan 9;6:20. doi: 10.1038/s43856-025-01234-6 (PMC12789550; doi:10.1038/s43856-025-01234-6)
Supplement: Supplementary file 4 — Supplementary Data 2 [file 43856_2025_1234_MOESM4_ESM.pdf]

Supplementary Data 2. Comparison of multi-modality studies

Signal[a]	Study	Anxiety induction[b]	Anxiety measurement[c]	Anxiety labeling approach	WD (body part)[d]	Sample size	Age [years][e]	Features[f]	Method[g]	Validation[h]	Result[i]
ECG & EDA	Jain & Kumar, 2024[1]	TSST	6-STAI	3-class (low/moderate/high) based on 6-STAI scores	RespiBAN (chest)	15 (M: 12, F: 3)	27.5 ± 2.4 (25–29)	ECG: Min ECG, Max ECG, Mean ECG, Var ECG, SD ECG, Mean HR, Mean RR, RMSSD, SDNN, TINN; EDA: Min EDA, Max EDA, Mean EDA, SD Eda, Mean Peak Duration SCR, Mean Peak Amp SCR	GBT	LOSO CV	ACC = 96.70%
ECG & EDA	Zhou et al., 2023[2]	Public speaking, bug-box tasks	SUDS	Binary (high vs. low anxiety) based on SUDS scores; scores ≥ 50 labeled as high, < 50 as low	Zephyr BioHarness 3.0 (chest, ECG); Grove-GSR Sensor (wrist, EDA)	52 (N/R)	N/R	ECG: Mean HR, RMSSD, SDNN, HF Power, LF Power, LF/HF, Mean ECG, Median ECG, SD ECG, Var ECG; EDA: Mean SCL, Rate SCR, Mean EDA, Median EDA, SD EDA, Var EDA	Ensemble (SVM, LGBM, RF, XGB)	5-fold CV	ACC = 64.50%
ECG & EDA	Zhou et al., 2023[2]	TSST	STAI	Binary (high vs. low anxiety) based on 6-item STAI scores; scores ≥ 15 labeled as high, < 15 as low	RespiBAN Professional (chest), Empatica E4 (wrist)	15 (M: 12, F: 3)	27.5 ± 2.4 (25–29)	ECG: Mean HR, RMSSD, SDNN, HF Power, LF Power, LF/HF, Mean ECG, Median ECG, SD ECG, Var ECG; EDA: Mean SCL, Rate SCR, Mean EDA, Median EDA, SD EDA, Var EDA	Ensemble (SVM, LGBM, RF, XGB)	5-fold CV	ACC = 99.00%
ECG & EDA	Ihmig et al., 2020[3]	VRET spider exposure	Self-reported anxiety level	Binary (anxious vs. non-anxious) based on subjective ratings and rest baseline	BITalino (both wrists, chest)	57 (N/R)	18–40	ECG: Mean HR, SD HR; EDA: Mean EDA, NFD EDA, Mean Peak Amp SCR, Nr of Peaks SCR	Bagged trees	10-fold CV	ACC = 89.80%
ECG & EDA	Gazi et al., 2021[4]	VRET spider exposure	Self-reported anxiety level	Binary (anxious vs. rest) based on exposure to spider clips vs. rest condition	BITalino (both wrists, chest)	55 (N/R)	18–40	ECG: Mean HR, SD HR, NFD HR, Mean RR, SDNN, RMSSD, pNN50, LF/HF, SD1/SD2, RSA; EDA: Mean SCL, NFD SCL, Nr of Peaks SCR, Mean Peak Amp SCR	RF	LOSO CV	ACC = 78.00%, F1 = 79.00%
EDA & PPG	Di Tecco et al., 2024[5]	Video	Self-reported predominant emotion	3-class (anxiety / other emotion / relaxation) based on self-reported dominant emotion per clip	Shimmer3 GSR+ (wrist)	32 (M: 19, F: 12)	19–28	TF domain: EDA: Ratio Max/Min SCL, Geometric Mean SCL; PPG: Mean HR, 3rd Quantile HR, MAD PPG, SD PPG ([3.5, 8] Hz), Occupied Bandwidth PPG ([0, 2] Hz), Mean Freq PPG ([0, 2] Hz, [2, 3.5] Hz, [0, 8] Hz). Deep domain: EDA ([0, 5] Hz), HR ([0, 2] Hz), PPG ([0, 8] Hz) via AlexNet relu3 layer.	Subspace KNN, Weighted KNN	Hold-out (train 90%, test 10%)	ACC = 95.22%, SE = 95.22%, PPV = 95.22%, F1 = 95.22%
EDA & PPG	Lee et al., 2020[6]	Video	Self-reported anxiety onsets	Binary (anxious vs. non-anxious) based on immediate self-report after each video	Empatica E4 (wrist)	23 (N/R)	23.3 ± 1.9	EDA: Mean EDA, SD EDA, Max EDA, Min EDA, Amp EDA; PPG: Mean Amp PPG, SD Amp PPG, Max Amp PPG, Min Amp PPG, Mean Dif PPI, SD Dif PPI, Length Dif PPI, Irregularity Dif PPI, Dif nPPI, Post Count Dif Fast PPI, LF/HF, CoV PPI	LR	LOTO CV	ACC = 49.26%
EDA & PPG	Petrescu et al., 2020[7]	VRET heights exposure	SUDS-10	3-class (low/mild/high) based on SUD scores	Shimmer3 GSR+ Uni	7 (N/R)	22–50	EDA: Mean SCR, MAR SCL, wAmp EDA, WAmp SCR; PPG: Mean HR, SD HR	Regression model	Hold-out (train 86%, test 14%)	ACC = 92.38%

(Table continued)

Signal[a]	Study	Anxiety induction[b]	Anxiety measurement[c]	Anxiety labeling approach	WD (body part)[d]	Sample size	Age [years][e]	Features[f]	Method[g]	Validation[h]	Result[i]
ECG, EDA & RSP	Gazi et al., 2021[4]	VRET spider exposure	Self-reported anxiety level	Binary (anxious vs. rest) based on exposure to spider clips vs. rest condition	BITalino (both wrists, chest)	55 (N/R)	18–40	ECG: Mean HR, SD HR, NFD HR, Mean RR, SDNN, RMSSD, pNN50, LF/HF, SD1/SD2, RSA; EDA: Mean SCL, NFD SCL, Nr of Peaks SCR, Mean Peak Amp SCR; RSP: Mean ReR, SD ReR, Mean IBI, SD IBI, RMSSD ReR, CoV ReR, ACF1 of ReR, CoV Ti, ACF1 Ti, RMSSD Ti, CoV Te, ACF1 Te, RMSSD Te, Mean Ti, Mean Te, Mean Ti/Te	RF	LOSO CV	ACC = 85.00%, F1= 95.22%
ECG, EDA & RSP	Khullar et al., 2021[8]	VRET spider exposure	Self-reported anxiety level	Binary (anxious vs. rest) based on exposure to spider clips vs. rest condition	BITalino (both wrists, chest)	57 (N/R)	18–40	N/R	ET	5-fold CV	ACC = 80.00%, SE = 80.00%, SP = 80.00%, F1 = 80.00%
EDA & RSP	Gazi et al., 2021[4]	VRET spider exposure	Self-reported anxiety level	Binary (anxious vs. rest) based on exposure to spider clips vs. rest condition	BITalino (both wrists, chest)	55 (N/R)	18–40	EDA: Mean SCL, NFD SCL, Nr of Peaks SCR, Mean Peak Amp SCR; RSP: Mean ReR, SD ReR, Mean IBI, SD IBI, RMSSD ReR, CoV ReR, ACF1 of ReR, CoV Ti, ACF1 Ti, RMSSD Ti, CoV Te, ACF1 Te, RMSSD Te, Mean Ti, Mean Te, Mean Ti/Te	RF	LOSO CV	ACC = 88.00%, F1 = 88.00%
EDA, PPG & SKT	Šalkevičius et al., 2019[9]	VRET public speaking	SUDS (0-100 scale)	Binary (anxious vs baseline) based on experimental stage timestamps	Empatica E4 (wrist)	30 (M: 17, F: 13)	27.5 ± 4.2 (21–34)	EDA: Min EDA, Max EDA, Mean EDA, Var EDA, SD EDA, Median EDA, Kurt EDA, Skew EDA, MAD EDA, 6th Mom EDA, 5th Mom EDA, 4th Mom EDA, 3rd Mom EDA, RMS EDA, 1st Dif EDA, 1st Dif/SD EDA, 2nd Dif EDA , 2nd Dif /SD EDA, MA Raw EDA, SD Raw EDA, MA 1st Dif Raw EDA, MA 1st Dif norm EDA, MA 2nd Dif EDA, MA 2nd Dif norm EDA, Nr of Peaks SCR, Mean Peak Amp SCR, Max Peak Amp SCR; PPG: Min PPG, Max PPG, Mean PPG, Var PPG, SD PPG, Median PPG, Kurt PPG, Skew PPG, MAD PPG, 6th Mom PPG, 5th Mom PPG, 4th Mom PPG, 3rd Mom PPG, RMS PPG, 1st Dif PPG, 1st Dif/SD PPG, 2nd Dif PPG , 2nd Dif /SD PPG, MA Raw PPG, SD Raw PPG, MA 1st Dif Raw PPG, MA 1st Dif norm PPG, MA 2nd Dif PPG, MA 2nd Dif norm PPG, Mean HR, MAD HR, RMSSD, SDNN, Mean PPI, SD PPI; SKT: Min SKT, Max SKT, Mean SKT, Var SKT, SD SKT, Median SKT, Kurt SKT, Skew SKT, MAD SKT, 6th Mom SKT, 5th Mom SKT, 4th Mom SKT, 3rd Mom SKT, RMS SKT, 1st Dif SKT, 1st Dif/SD SKT, 2nd Dif SKT , 2nd Dif /SD SKT, MA Raw SKT, SD Raw SKT, MA 1st Dif Raw SKT, MA 1st Dif Norm SKT, MA 2nd Dif SKT, MA 2nd Dif norm SKT	SVM	10-fold CV	ACC = 86.30%

(Table continued)

Signal[a]	Study	Anxiety induction[b]	Anxiety measurement[c]	Anxiety labeling approach	WD (body part)[d]	Sample size	Age [years][e]	Features[f]	Method[g]	Validation[h]	Result[i]
EDA, PPG & SKT	Shaukat-Jali et al., 2021[10]	Impromptu speech task	LSAS, SPSQ	4-class (low/mild/moderate/high) based on SUDS score.	Empatica E4 (wrist)	12 (M: 5, F: 7)	19 ± 1.8	N/R	KNN	10-fold CV	ACC = 99.48%
ECG, EDA & EMG	Vaz et al., 2023[11]	TSST	6-STAI	Binary (low/moderate vs high anxiety) based on 6-STAI scores	RespiBAN (chest) and Empatica E4 (wrist)	15 15 (M: 12, F: 3)	27.5 ± 2.4 (25–29)	ECG: AUCC, Range ECG, Max ECG, Var ECG, Median ECG, Mean RR, MAD RR, TINN, pNN50, Log HF Power, VHF Power, LF Norm, Min HR, Max HR, Range HR, SDTT, Min ECG, Var HR; EDA: Min EDA, Median SCR, Max Peak Amp SCR, Corr SCR, SCR Rate, Median Peak Amp SCR, Median SCR Onsets Amp, Median SCR Recovery Amp, Mean SCR Recovery Amp, Median Recovery Time, Mean Recovery Time, Max SCL, Median SCL; EMG: Med EMG, Perc 10 EMG, Perc 10 Env, Nr Pulse Onsets/sec, Nr Activ Peaks/sec, Activ Peaks Mean Amp, Activ Peaks Med Amp, Pulse Onsets Med Amp	Adaptive boosting	5-fold CV	ACC = 92.00%, SE = 86.70%, PPV = 87.50%, F1 = 86.40%
ECG & RSP	Gazi et al., 2021[4]	VRET spider exposure	Self-reported anxiety level	Binary (anxious vs. rest) based on exposure to spider clips vs. rest condition	BITalino (both wrists, chest)	55 (N/R)	18–40	ECG: Mean HR, SD HR, NFD HR, Mean RR, SDNN, RMSSD, pNN50, LF/HF, SD1/SD2, RSA; RSP: Mean ReR, SD ReR, Mean IBI, SD IBI, RMSSD ReR, CoV ReR, ACF1 of ReR, CoV Ti, ACF1 Ti, RMSSD Ti, CoV Te, ACF1 Te, RMSSD Te, Mean Ti, Mean Te, Mean Ti/Te	RF	LOSO CV	ACC = 68.00%, F1 = 71.00%
ECG & PPG	Sinche et al., 2024[12]	Pre- and post-test evaluation	Cognitive Test Anxiety Scale	Binary (anxious vs. non-anxious) based on pre-test (anxious) vs. post-test (non-anxious) phases	AD8232 (chest), MAX30100 (finger)	20 (M: 18, F: 2)	19–27	ECG: Mean HR, SDNN, RMSSD, pNN50, SDSD; PPG: SDNN, RMSSD, pNN50, Mean HR, Mean PPI; PAT: Mean PAT, SD PAT, SD PATV, RMS PATV	SVM	10-fold CV	ACC = 99.84%
PPG & SKT	Bao et al., 2024[13]	Rest and relaxation with biofeedback	STAI	3-class (relaxed/mild/severe) based on STAI score	Empatica E4 (wrist)	20 (M: 0, F: 20)	29.1 ± 3.6	PPG: Mean HR, Mean PPG, SD PPG, MSE PPG, LF Power PPG, MF Power PPG, HF Power PPG, Spectral Power Ratio PPG, Tachogram Spectral Values, Tachogram Spectral Ratio, SDNN, LF/HF, RMSSD, PNSi, SNSi; SKT: Mean, SD, Kurt, Skew, Spectral Power	SVM	LOSO CV	F1 = 69.30%

Notes:

[a] ECG = electrocardiogram, EDA = electrodermal activity, EMG = electromyography, PPG = photoplethysmogram, RSP = respiratory signal, SKT = skin temperature.

[b] TSST = Trier Social Stress Test, VRET = Virtual Reality Exposure Therapy.

[c] 6-STAI = Six-Item State-Trait Anxiety Inventory, SUDS = Subjective Units of Distress Scale

[d] WD = wearable device.

[e] F = female, M = male.

[f] HR = heart rate, SD = standard deviation NFD = normalized first difference, RR = R-R interval (time between successive R waves in an ECG signal), SDNN = standard deviation of normal-to-normal R-R intervals (or pulse peak intervals (PPI) in PPG), RMSSD = root mean square of successive differences of R-R intervals (or PPI in PPG), pNN50 = percentage of successive R-R intervals (or PPI) differing by more than 50 ms, LF = low frequency, HF = high frequency, SD1SD2 = Poincaré plot features: short, term (SD1) and long-term (SD2) variability, RSA = respiratory sinus arrhythmia, SCL = skin conductance level (tonic component of EDA), Nr = number, Max = maximum, TF = time and frequency domain, Min = minimum, MAD = mean absolute deviation, Freq = frequency, Amp = amplitude, Dif = difference, PPI = pulse peak interval, CoV = coefficient of variation, SCR = skin conductance response (phasic component of EDA), wAmp = weighted

amplitude, ReR = respiratory rate, IBI = inter-breath interval, ACF1 = first-lag autocorrelation, Ti = inspiratory time, Te = expiratory time, Kurt = kurtosis, Skew = skewness, Mom = moment (3rd, 4th, 5th, or 6th moment of the signal distribution), MA = mean absolute value, RMS = root mean square, Var = variance, AUCC = area under the correlation curve, Norm = normalized, SDTT = standard deviation of total time, PNSi = parasympathetic nervous system index, SNSi = sympathetic nervous system index.

[g] GBT = gradient boosted trees, SVM = support vector machine, LGBM = light gradient boosting machine, XGB = extreme gradient boosting, RF = random forest, KNN = k-nearest neighbors, LR = logistic regression, ET = extra trees.

[h] LOSO = leave-one-subject-out, CV = cross-validation, LOTO = leave-one-trial-out.

[i] ACC = accuracy, F1 = F1-score, PPV = precision, SE = sensitivity, SP = specificity.

References

1. Jain, A. & Kumar, R. Machine learning based anxiety detection using physiological signals and context features. In 2024 2nd International Conference on Advancement in Computation & Computer Technologies (InCACCT), 116–121, DOI: 10.1109/InCACCT61598.2024.10551003 (2024).
2. Zhou, E., Soleymani, M. & Matarić, M. J. Investigating the generalizability of physiological characteristics of anxiety. In 2023 IEEE International Conference on Bioinformatics and Biomedicine (BIBM), 4848–4855, DOI: 10.1109/BIBM58861.2023.10385292 (2023).
3. Ihmig, F. R., Neurohr-Parakenings, F., Schäfer, S. K., Lass-Hennemann, J. & Michael, T. On-line anxiety level detection from biosignals: Machine learning based on a randomized controlled trial with spider-fearful individuals. PLoS One 15, e0231517, DOI: 10.1371/journal.pone.0231517 (2020).
4. Gazi, A. H. et al. Respiratory markers significantly enhance anxiety detection using multimodal physiological sensing. In 2021 IEEE EMBS International Conference on Biomedical and Health Informatics (BHI), 1–4, DOI: 10.1109/BHI50953.2021.9508589 (Athens, Greece, 2021).
5. Di Tecco, A., Pistolesi, F. & Lazzerini, B. Elicitation of anxiety without time pressure and its detection using physiological signals and artificial intelligence: A proof of concept. IEEE Access 12, 22376–22393, DOI: 10.1109/ACCESS.2024.3362668 (2024).
6. Lee, S., Lee, T., Yang, T., Yoon, C. & Kim, S.-P. Detection of drivers' anxiety invoked by driving situations using multimodal biosignals. Processes 8, DOI: 10.3390/pr8020155 (2020).
7. Petrescu, L. et al. Integrating biosignals measurement in virtual reality environments for anxiety detection. Sensors 20, DOI: 10.3390/s20247088 (2020).
8. Khullar, V., Tiwari, R. G., Agarwal, A. K. & Dutta, S. Physiological signals based anxiety detection using ensemble machine learning. In Tavares, J. M. R. S., Dutta, P., Dutta, S. & Samanta, D. (eds.) Cyber Intelligence and Information Retrieval, 597–608, DOI: 10.1007/978-981-16-4284-5_53 (Springer Singapore, Singapore, 2022).
9. Šalkevicius, J., Damaševičius, R., Maskeliunas, R. & Laukienė, I. Anxiety level recognition for virtual reality therapy system using physiological signals. Electronics 8, DOI: 10.3390/electronics8091039 (2019).
10. Shaukat-Jali, R., van Zalk, N. & Boyle, D. Detecting subclinical social anxiety using physiological data from a wrist-worn wearable: Small-scale feasibility study. JMIR Form. Res. 5, e32656, DOI: 10.2196/32656 (2021).
11. Vaz, M., Summavielle, T., Sebastião, R. & Ribeiro, R. P. Multimodal classification of anxiety based on physiological signals. Appl. Sci. 13, DOI: 10.3390/app13116368 (2023).
12. Sinche, S., Acán, J. & Hidalgo, P. Anxiety detection using consumer heart rate sensors. Eng. Proc. 77, DOI: 10.3390/engproc2024077010 (2024).
13. Bao, Y., Xue, M., Gohumpu, J. et al. Prenatal anxiety recognition model integrating multimodal physiological signal. Sci. Reports 14, 21767, DOI: 10.1038/s41598-024-72507-8 (2024).
